# Minimizing Redundancy in Wireless Sensor Networks Using Sparse Vectors

**DOI:** 10.3390/s25051557

**Published:** 2025-03-03

**Authors:** Huiying Yuan, Cuifang Gao

**Affiliations:** School of Science, Jiangnan University, Wuxi 214122, China; yuanhuiying2022@163.com

**Keywords:** wireless sensor networks, sparse vector compression, temporal and spatial similarity, sample rate adjustment

## Abstract

In wireless sensor networks, sensors often collect and transmit a large amount of redundant data, which can lead to excessive battery consumption and subsequent performance degradation. To solve this problem, this paper proposes a Zoom-In Zoom-Out (ZIZO) method based on sparse vectors (SV-ZIZO). It operates in two parts: At the sensor level, given the temporal similarity of the data, a new compression method based on the sparse vector representation of segmented regions is proposed. This method can not only effectively ensure the compression ratio but also improve the accuracy of data restoration. At the cluster-head (CH) level, by utilizing the spatial similarity of the data, the fuzzy clustering theory is introduced to put some sensors into hibernation mode, thereby reducing data transmission. Meanwhile, the sampling frequency of the sensors is dynamically adjusted by calculating the redundancy rate of the collected periodic data. The experimental results show that compared with other existing methods, the algorithm proposed in this paper increases the data compression ratio by 21.8% and can reduce energy consumption by up to 95%.

## 1. Introduction

Wireless sensor networks (WSNs) [1] usually consist of a large number of sensor nodes with communication capabilities, mostly with battery-powered solutions, small in size, and limited in resources. They are generally densely deployed to monitor their surroundings and send data to an external receiver or base station. WSNs are widely used in the fields of habitat monitoring [2], environmental monitoring [3], and healthcare monitoring [4], among others. These nodes are often equipped with minimal communication, computation, storage, and battery resources. In complex environments, replenishing the sensors with power consumes a lot of resources, so reducing the energy consumption of sensors and increasing the duration of their use is particularly important in the design of wireless sensor networks.

Due to factors such as sensor installation location and the frequency of collection, the data generate a large amount of redundancy in the temporal as well as spatial dimensions [5]. The transmission of these redundant data creates multiple problems for the network such as bandwidth consumption, energy consumption, and multiple associated overhead costs such as data storage, processing, and communication. In wireless sensor networks, data transmission is the highest energy-consuming process for sensor nodes compared to data processing [6]. Therefore, the main way to save the energy of sensor nodes is to reduce data collection and transmission [7]. Data fusion as well as tuning the sensor sampling frequency are important techniques to reduce data collection and transmission.

For the fusion and recovery of periodic data, PIP-DA [8] uses distance metrics to exploit the temporal correlation of data and aggregates data at the sensor level. However, the data recovery accuracy is affected by the distance metric and the complexity of the data. PFF [9] is a prefix filtering method that achieves relatively high data recovery accuracy, yet the data compression ratio depends on the accuracy of the “aggregate similarity function”. ATP [10] searches for temporal similarity in cycle-based data during the aggregation phase to eliminate redundancy. In the transmission phase, it employs a one-way ANOVA model and the F-test to identify the correlations between data cycles and determine whether to upload data. However, both the compression ratio and data accuracy are highly correlated with the division of cycles. EDaTAP [11] considers the similarity between sets of unequal-length measurements from different sensors within the same cycle, which improves the compression of complex data to some extent, but it is difficult to guarantee the recovery accuracy of unequal-length data. EK-means [12] applies Euclidean-metric-based techniques to eliminate redundant data and, at the cluster-head level, uses an enhanced K-means algorithm to group similar datasets generated by neighboring nodes into the same clusters. However, the energy consumption of data transmission at the sensor level still needs to be further reduced.

For the adjustment of data sampling frequency, researchers have put forward various acquisition-frequency adjustment methods to cut down power consumption. ASMS [13] is an energy-aware adaptive sampling algorithm that sets a lower sampling rate and ensures self-sustainability when the residual energy of the sensors is too low. ASIA [14] is a method that considers adaptive sampling interval adjustment based on a dual-input single-output fuzzy logic controller. DDASA [15] is an adaptive power management method. EDSAS [16] minimizes the sensor sampling rate by minimizing the mean squared error between the estimated samples and the actual signal values to estimate the future values with high accuracy. To ensure the long-term operation of the network, Harb et al. [17] proposed three mechanisms to adapt the sensors to the changes in their sampling rate based on the similarity function, distance function, and statistical test ANOVA. ZIZO [18] implements IBE compression at the sensor level and inverse adjustment of the redundancy rate based on the temporal correlation at the CH level.

Although existing algorithms have achieved certain results in energy conservation at the sensor level and the CH level, it is difficult for them to ensure data recovery accuracy while reducing the data volume. This paper focuses on the challenging issue of energy-saving management in wireless sensor networks and innovatively proposes an adjustment mechanism based on the cluster network structure. This mechanism consists of two parts applicable to the sensor level and the CH level.

- At the sensor level, this paper presents an improved data-compression method. This method combines segmented regions with quartile screening to precisely locate the corresponding key values and then performs sparse vector compression. By deeply exploring the similarity between each segment of data and its corresponding key value, the amount of transmitted data can be significantly reduced.
- At the CH level, this paper optimizes the reverse sampling rate adjustment method. By fully leveraging the spatial correlation among the data of different sensor nodes, a sensor sleep decision is set. This decision can dynamically adjust the sampling frequency of active sensors within the cluster in the next cycle according to the actual network situation, effectively balancing the accuracy of data collection and energy consumption and providing a more efficient solution for energy-saving management at the CH level.

## 2. Methods

### 2.1. Network Model Overview

**Figure 1:**
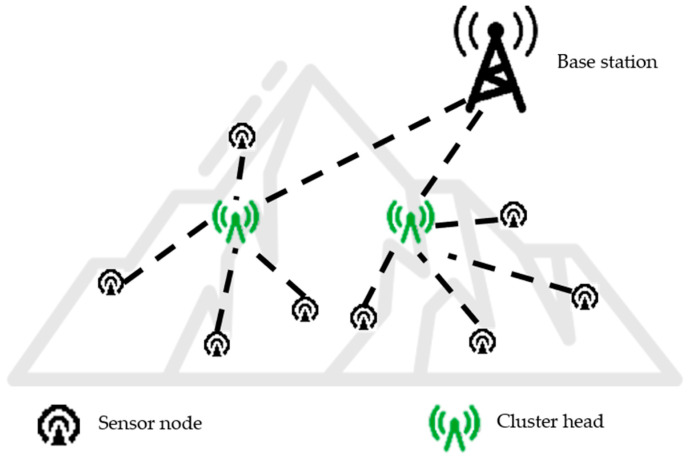
Cluster-based periodic network architecture.

The cluster-based periodic network architecture is a common configuration in wireless sensor networks. Its fundamental composition, as presented in Figure 1, typically consists of three elements: sensor nodes, cluster heads, and base stations.

To ensure the connectivity of the network, the sensors are organized as a connected graph *G* = (*S*, *A*), where $S=\{S_{1},S_{2},\ldots ,S_{n}\}$ is the set of sensor nodes and *A* is the set of arcs connecting the nodes. In a periodic network, the data collected by each sensor $S_{i}$ in the *p*-th cycle are represented as a vector $S_{i}^{p}$, defined as follows:

(1) $$S_{i}^{p}=[s_{\left(i,1\right)},\;s_{\left(i,2\right)},\;\ldots ,\;s_{\left(i,\tau \right)}]$$

where *τ* reflects the total number of data obtained in cycle *p*. The data matrix for each cluster collected by CH is represented as follows:

(2) $$S^{p}=\left[\begin{array}{ccc} s_{\left(1,1\right)} & \cdots & s_{(1,\tau )}\\ \vdots & \ddots & \vdots \\ s_{(n,1)} & \cdots & s_{\left(n,\tau \right)} \end{array}\right]$$

The reduction and amplification algorithm proposed in this paper mainly undertakes the compression and uploading of $S_{i}^{p}$ at the sensor node. Subsequently, at the CH, it performs the decompression and amplification of $S^{p}$ and adjusts the sampling frequency. This method enables the reduction of the collected data volume while ensuring the accuracy of data collection.

### 2.2. Minimizing Data Redundancy Algorithm Based on Segmented Sparse Vectors (SV-ZIZO Algorithm)

#### 2.2.1. Indexed Bit Coding Compression Algorithm

The existing ZIZO algorithm [18] is composed of two parts. In this section, the first part is discussed initially. Specifically, before the data are transmitted to the CH, the sensor node compresses them using the Indexed Bit Encoding algorithm (IBE). The core element of the IBE algorithm is conducting a key-value $u_{\left(i,j\right)}$ search for the captured data sequence $S_{i}^{p}$, where the value *j* represents the *j*-th key value. Subsequently, the algorithm calculates the index bit information that satisfies $\Delta \left(u_{\left(i,j\right)},s_{\left(i,k\right)}\right)\leq \varepsilon$ by using a given threshold and records it in the form of 0–1 similarity coding. That is, similarity is denoted as 1 and dissimilarity as 0. Here, $\Delta \left(u_{\left(i,j\right)},s_{\left(i,k\right)}\right)$ is defined as follows:

(3) $$\Delta \left(u_{\left(i,j\right)},\;s_{\left(i,k\right)}\right)=\left|u_{\left(i,j\right)}-s_{\left(i,k\right)}\right|\;(k=1,2,\ldots ,\tau )$$

Meanwhile, the coding method is shown in Equation (4) as follows:

(4) $${Code}_{\left(j,k\right)}=\left\{\begin{array}{cc} 1 & \Delta \left(u_{\left(i,j\right)},s_{\left(i,k\right)}\right)\leq \varepsilon \\ 0 & \Delta \left(u_{\left(i,j\right)},s_{\left(i,k\right)}\right)>\varepsilon \;\mathrm{or}\;s_{\left(i,k\right)}=null \end{array}\right.$$

By comparing $u_{\left(i,j\right)}$ with the data in $S_{i}^{p}$ one by one, a 0–1 vector ${code}_{j}$ with a length of p can be obtained. Finally, using the binary-to-decimal conversion method, the binary ${\mathrm{code}}_{j}$ is encoded into a decimal $c_{j}$, and a key data pair $\left(u_{\left(i,j\right)},c_{j}\right)$ is formed with $u_{\left(i,j\right)}$. When the search for all key data in $S_{i}^{p}$ is completed, $S_{i}^{p}$ can be compressed into the vector $U_{i}^{p}$ which is defined as follows:

(5) $$U_{i}^{p}=\left\{\left(u_{\left(i,1\right)},c_{1}\right),\;\left(u_{\left(i,2\right)},c_{2}\right),\ldots ,(u_{\left(i,j\right)},c_{j})\right\}$$

where the key value $u_{\left(i,j\right)}$ of the existing IBE algorithms is computed as described in the literature [18].

#### 2.2.2. Compression Method Based on Segmented Sparse Vector Representation

At the sensor-node network layer, the existing ZIZO algorithm is capable of reducing the data volume to a certain degree by leveraging the temporal correlation of the data, and it exhibits a relatively favorable compression ratio. Nevertheless, the real-time compression implemented in the existing ZIZO algorithm introduces certain uncertainties in the data decompression process. Thus, based on the periodic network architecture, this paper puts forward a compression approach founded on the representation of segmented-region sparse vectors. Through the strategy of first collecting data periodically and then processing them, this method not only alleviates the CPU load but also further compresses the data, thereby minimizing the transmission of time redundant data.

This paper takes into account the physical characteristics of the sensors during data collection. That is, the sensors will remain relatively stable within a certain period of time, but there are cases where this stable duration is shorter than the upload cycle. Therefore, for the data $S_{i}^{p}$ of the *i*-th sensor in the *p*-th cycle, the algorithm proposed in this paper considers processing them in a segmented form. Specifically, $S_{i}^{p}$ is divided into several segments as follows:

(6) $$S_{i}^{p}=S_{i}^{p\left(1\right)}\cup S_{i}^{p\left(2\right)}\cup \ldots {\cup S}_{i}^{p\left(v\right)}\cup \ldots {\cup S}_{i}^{p\left(V\right)}$$

Here, *v* serves as the segmentation counter, and the total number of segments is *V*. The value of *V* is determined by the characteristics of the dataset and the number of data points *τ* within the selected cycle.

After segmentation, key-value searches are performed for each segment of data. Given the various situations that may occur in the segmented data, such as overall data similarity, head-part data similarity, or tail-part data similarity, this paper takes into account the quartile set $Q_{v}$ of each data segment. The key value $u_{\left(i,j\right)}^{v}$ is then selected from $Q_{v}$. During the selection process, the mean feature of each data segment, denoted as ${\overset{–}{m}}^{p\left(v\right)}$, is incorporated. Denote $Q_{v}=\left\{Q_{25\%}^{p\left(v\right)},Q_{50\%}^{p(v)},Q_{75\%}^{p(v)}\right\}$, which are, respectively, the first quartile, the second quartile, and the third quartile of each segment of data $S_{i}^{p(v)}$. The selection conditions for the key value of each segment are satisfied as follows:

(7) $$u_{(i,1)}^{v}=select\left(\min\left(\left|Q_{25\%}^{p\left(v\right)}-{\overset{–}{m}}^{p\left(v\right)}\right|,\left|Q_{50\%}^{p\left(v\right)}-{\overset{–}{m}}^{p\left(v\right)}\right|,\left|Q_{75\%}^{p\left(v\right)}-{\overset{–}{m}}^{p\left(v\right)}\right|\right)\right)$$

Here, *select* is a selection function, and its return value is the value in $Q_{m}$ that is closest to ${\overset{–}{m}}^{p\left(m\right)}$. $u_{(i,1)}^{v}$ is the first key value obtained through the search, which represents the vast majority of the values in this segment of data. Using the mean and quartiles in Equation (7) can make the selected key values closer to the key positions of the data. Because outliers in the data can affect the mean value of the data, this method can identify the sudden changes in the data after cleaning.

Substituting the key value into Equation (3), the result is as follows:

(8) $$\Delta (u_{\left(i,1\right)}^{v},s_{\left(i,k\right)}^{p(v)})=\left|s_{\left(i,k\right)}^{p(v)}-u_{\left(i,1\right)}^{v}\right|$$

At the same time, by referring to the IBE method and utilizing the custom threshold *ε*, 0–1 similarity encoding is performed through Equation (4). At this time, most of the values in $S_{i}^{p(v)}$ can be compressed by $u_{\left(i,1\right)}^{v}$. For the remaining data that have not been compressed, the algorithm directly records their data values and position indices. Thus, the vector of Equation (5) within each segment is shown as follows:

(9) $$U_{i}^{p(v)}=\left\{\left(u_{(i,1)}^{v},c_{1}\right),\;\left(s_{\left(i,k\right)}^{p(v)},k\right),\ldots \right\}$$

The amount of data after the compression of $U_{i}^{p(v)}$ depends on the amount of data that has not been compressed by $Q_{v}$. Due to the characteristic that the differences in data within adjacent short time intervals are not significant, the more similar the sensor data in the p-period to the key values of their corresponding segment, the higher the temporal redundancy of the data in this period. Therefore, the overall compression ratio of the algorithm is relatively good.

In this paper, the compression algorithm based on the segmented-region sparse vector representation (Algorithm 1) mainly consists of two parts. The first part is the segmentation function (Data-Split function), which is responsible for dividing the data into *V* data segments. The pseudocode algorithm is shown as follows:
**Algorithm 1:** Data-Split function pseudocode01 Read vector Si_p, int V 02 Set vector si_p_v 03 len_v = floor(length(Si_p)/V) 04 for v = 0 to V 05 for i = 1 to len_v 06    if Si_p(v∗len_v + i) ~= NaN 07      si_p_v(i)=Si_p(v∗len_v + i) 08 Return si_p_v

In the algorithm, floor is the floor function, length represents the length or is a function. Both are based on MATLAB syntax, and the following algorithm is also based on MATLAB syntax. The function returns si_p_v, which is the segmented data and serves as the input for the following algorithm. Based on the result of si_p_v, the second part of the SV-ZIZO algorithm in this paper is the calculation of key values based on quartiles and compression, which is presented as Algorithm 2:

**Algorithm 2:** Key-value calculation and compression based on quartiles01 Read vector si_p_v, int ε 02 Set vector U 03 Q = [prctile(si_p_v, 25), prctile(si_p_v, 50), prctile(si_p_v, 75)] 04 m = mean(si_p_v) 05 index = min(abs(Q[1]-m), abs(Q[2]-m), abs(Q[3]-m))[2] 06 u_p_v = Q[index] 07 len_p_v = length(si_p_v) 08 Set code = zeros(len_p_v) 09 for i = 1 to len_p_v 10   if abs(si_p_v[i] − u_p_v) < ε 11     code[i] = 1 12     si_p_v[i] = NaN 13   else 14     code[i] = 0 15 c = HexToDec(code) 16 u(1) = (u_p_v, c) 17 append(U, u(1)) 18 while Si_p is all NaN 19   Set code = zeros(len_p_v) 20   u = min(si_p_v) 21   for k = 1 to len_p_v 22     if | u_i − Si_p(k)| <= ε 23       code(k) = 1 24       si_p_v[i] = NaN 25     else 26       code(k) = 0; 27   c = HexToDec(code) 28   u = (u_i, c) 29   append(U, u) 30 Return U

In the algorithm, NaN represents a non-number and cannot participate in the calculation of the minimum value, min is the function for calculating the minimum value, HexToDec is the function for converting binary to decimal, and append is the function for inserting elements into a vector. The algorithm finally returns the compressed dataset. By merging the data of each segment, the compression results of $S_{i}^{p}$ are calculated in the end.

The primary goal of data aggregation is to eliminate redundancy in data transmission, reduce communication load, and, thus, lower energy consumption. If the temporal redundancy of data is high within a short period, using the quartile adjustment method based on segmented regions can not only ensure the flexibility of segmentation but also guarantee that the size of the compressed reading set is much smaller than that of the original reading set.

#### 2.2.3. Sensor Sampling Rate Adjustment Method at the CH Level

At the sensor level, this paper employs a key-value-based data compression algorithm to reduce energy consumption during wireless transmission by minimizing data volume. At the CH level, we leverage transmitted, aggregated, and decompressed environmental data to comprehensively analyze feature relationships among data, identify potentially similar sensors, and implement sensor operations at the CH level through two steps—dormancy of similar sensors and sampling rate adjustment—thereby achieving energy efficiency.

First, the data collected, compressed at the sensor level, and uploaded to the CH are decompressed. They are mainly divided into two parts. The first part is $\left(u_{(i,1)}^{v},c_{1}\right)$. In this part, most of the values in this data segment are restored to the key values. The other part is $\left(s_{\left(i,k\right)}^{p(v)},k\right)$. According to the position index k, a small portion of the detailed data $s_{\left(i,k\right)}^{p(v)}$ is restored and placed at the corresponding position in the original data segment. The processed data segments $U_{i}^{p(v)}$ are integrated in the original order, thus obtaining the complete sensor data. That is $S_{i}^{p}\prime =\left(s_{\left(i,1\right)}^{\prime },{\;s}_{\left(i,2\right)}^{\prime },\ldots ,{\;s}_{\left(i,\tau \right)}^{\prime }\right)$, and at this point, the decompression process is completed.

For the above decompression process, there is a recovery accuracy range, which is affected by the compression threshold ε. The specific range is as follows: When ε is less than the minimum value of the difference between the key value u and any data within the cycle, the data will not be compressed at this time, and their recovery accuracy is 100%. When ε is greater than the maximum value of the difference between the key value $u_{(i,1)}^{v}$ and any data within the cycle, the compression ratio reaches the maximum at this time, and the recovery accuracy reaches the minimum, which is $\frac{num(S_{i}^{p(v)}==u_{(i,1)}^{v})}{num(S_{i}^{p(v)})}$, and *num* is a function used to count the quantity of values that meet the conditions. The specific value for evaluating the recovery accuracy is shown in Equation (14), which will evaluate the decompression effect in detail.

This paper represents the data matrix composed of all cluster members at the CH level as follows:

(10) $${S^{p}}^{\prime }=\left(\begin{array}{ccc} s_{\left(1,1\right)}^{\prime } & \cdots & s_{(1,\tau )}^{\prime }\\ \vdots & \ddots & \vdots \\ s_{(n,1)}^{\prime } & \cdots & s_{\left(n,\tau \right)}^{\prime } \end{array}\right)$$

Starting from the data, the fuzzy clustering algorithm [19] is used to explore potential similar sensors. The fuzzy clustering algorithm can, according to specific parameters, cluster data with unclear boundaries and objects with similar characteristics, namely, the sensors in this paper, into multiple groups.

Because the fuzzy clustering algorithm is a well-established algorithm, this paper will not elaborate on it. In the calculation of the fuzzy relation matrix, the max–min method [20] is adopted. Then, classification is carried out according to the fuzzy similarity matrix using different confidence levels *λ* ∈ [0, 1]. Meanwhile, an appropriate λ value is selected based on the F-statistic [19]. The larger the value of the F-statistic, the more obvious the differences between classes. Here, the selection of *λ* [19] is related to the significance level *p*-value of the F-statistic corresponding to this value. When the *p*-value is less than a certain significance-level threshold (it is generally chosen as 0.05) and the F-statistic is large, the classification at this time is considered reasonable. Then, this *λ* is selected, and an appropriate classification $S=S_{1}\cup S_{2}\cup \ldots \cup S_{k}$ is determined, where *k* is the number of classes.

After the fuzzy clustering algorithm is completed, for each class in which the number of sensors is greater than 2, this paper considers selecting an edge sensor in this class to keep it dormant. The selection criterion is that for each eligible $S_{k}$, $\min err=\sum_{j=1}^{\tau }{\left(\bar{S_{\mathrm{kj}}^{1}\;}-\bar{S_{j}^{1}}\right)}^{2}$. Here, $S_{\mathrm{kj}}^{1}$ represents the data mean of class $S_{k}$ after removing the edge sensor, and $\bar{S_{j}^{1}}$ represents the original data mean of this class. By minimizing the mean difference, the class $S_{k}^{\prime }$ after removing the edge sensor is obtained. Eventually, $S^{\prime }=S_{1}^{\prime }\cup S_{2}^{\prime }\cup \cdots \cup S_{k}^{\prime }$ can be obtained, where the total number *l* of sensors contained in $S^{\prime }$ satisfies *l ≤ n*.

Next, consider the redundancy rate (RR) of $S^{\prime }$, which refers to the similarity of data at adjacent time points within the same cluster. In $S^{\prime }$, starting from whether the data vectors $S_{t+1}=\left(s_{(1,t+1)}^{\prime },s_{(2,t+1)}^{\prime },\ldots ,s_{(l,t+1)}^{\prime }\right)$ and $S_{t}=\left(s_{(1,t)}^{\prime },\;s_{(2,t\;)}^{\prime },\;\ldots ,\;s_{\left(l,t\right)}^{\prime }\right)\left(1\;\leq t\;\leq \tau -\;1,\;t\in N^{+}\right)$ at two adjacent time points are similar, the *T*-test is used to calculate the redundancy rate of this cycle. The null hypothesis of the *T*-test is that there is no significant difference between the data vectors at two adjacent time points, and the alternative hypothesis is that there is a significant difference between them. The calculation formula of the *T*-test is as follows:

(11) $$T=\frac{\bar{\left(S_{t+1}-S_{t}\right)}-\mu }{\frac{\sigma_{t,t+1}}{\sqrt{l}}}$$

where $\bar{S_{t+1}-S_{t}}$ is the average of the differences between the data vectors of two adjacent time instants $S_{t+1}$ and $S_{t}$, *μ* is the mean of all data, and $\sigma_{t,t+1}$ is the standard deviation of the differences between $S_{t+1}$ and $S_{t}$.

When conducting the *T*-test, a *T*-test value *t* is chosen that corresponds to a certain expected probability of false rejection α∈[0,1].

Finally, the redundancy rate of this period *p* is calculated using Equation (12), which is expressed as the ratio of the total number of similar time instants to the number of similarity calculations. Equation (12) [18] is shown as follows:

(12) $$\mathrm{RR}=\frac{\sum_{t=1}^{\tau -1}T\leq t}{\tau \;-\;1}$$

Calculate the redundancy rate of the data collected by this CH in each cycle according to the above formula, and inversely adjust the sampling frequency of all sensors in each group in the next cycle. Because the choice of sampling frequency affects the amount of collected data, which further leads to some loss of data information, the range of frequency adjustment is shown in Table 1.

#### 2.2.4. Complexity Analysis

In this section, we analyze the complexity of the algorithm from two aspects: the sensor level and the CH level.

First, at the sensor level, there are two core components: data segmentation and key-value extraction and compression. The time complexity of the data segmentation function is O(N), and the time complexity of the key-value calculation and compression function for each segment is O(len_p_v²). If all segments have approximately the same length L and the number of segments is V, satisfying N = V × L, then the overall time complexity is mainly determined by the key-value calculation and compression, which is O(N × L). In the worst-case scenario, when L approaches N, the time complexity approaches O(N²). Here, N is the total amount of input data from a single sensor.

At the CH level, the time complexity of fuzzy clustering is O(n × k × d × t), where n is the number of sensors, k is the number of clusters, d is the data dimension, and t is the number of iterations. For each class containing m sensors, calculating the mean squared error to select the edge sensors requires O(m²) operations. If there are k classes, the total complexity becomes O(k × m²). After putting the edge sensors into the dormant state, for τ − 1 adjacent time intervals, it is necessary to analyze the redundancy rate of the remaining n′ sensors. The time complexity of redundancy rate calculation is O(n′ × τ − 1), and the complexity of sampling rate adjustment is O(n′).

Therefore, the overall time complexity of the method proposed in this paper is O(N × L)+O(n × k × d × t) +O(k × m²)+O(n′ × TAO-1)+O(n′).

## 3. Results

In this paper, the improved algorithm is used to conduct experiments on the simulated IBRL dataset [21] and the LUCE dataset [21] in the real environment. First, at the sensor-node level, the compression algorithm based on the segmented-region sparse vector representation (Algorithm 1) and the IBE algorithm are evaluated in terms of compression ratio and mean squared error. Second, at the CH level, the reverse sampling rate adjustment algorithm based on fuzzy theory is compared with the original sampling rate adjustment algorithm in terms of energy consumption, and the change in redundancy rate after different sampling rate adjustments by the improved method is presented. The experiment was implemented in MATLAB R2023a, and the operating environment is a CPU of Intel Core i7-6700HQ, with 8 GB of memory and Windows 10 22H2.

### 3.1. Parameter Settings and Evaluation Indicators

Table 2 shows the values of the simulation parameters. In order to make a comparison with other methods, this paper selects cycles of 32 or 64 to ensure that comparisons can be made under the same conditions with other methods to evaluate the performance of the SV-ZIZO algorithm.

Next, we present the evaluation indicators selected in this paper and their corresponding calculation formulas.

1. The calculation formula of the evaluation metric Compressed Ratio (*CR*) is as follows:
  (13) $$\mathrm{CR}=\frac{len(Compressed\;Data)}{len(Original\;Data)}$$
2. Calculate the average error between the reconstructed data ${S_{i}^{p}}^{\prime }$ of the *i*-th sensor within one cycle and the original collected data $S_{i}^{p}$ of the corresponding sensor. The calculation formula is as follows:
  (14) $${MSE}_{i}=\frac{1}{\tau }\sum {\left({S_{i}^{p}}^{\prime }\;-\;S_{i}^{p}\right)}^{2}$$

### 3.2. IBRL Dataset and Result Analysis

The IBRL dataset was collected using a wireless sensor network (WSN) deployed in the Intel Laboratory at the University of Berkeley. This wireless sensor network consists of 54 Mica2Dot sensor nodes and records experimental data for 38 days from 28 February to 5 April 2004, with a sampling interval of 31 s.

**Figure 2:**
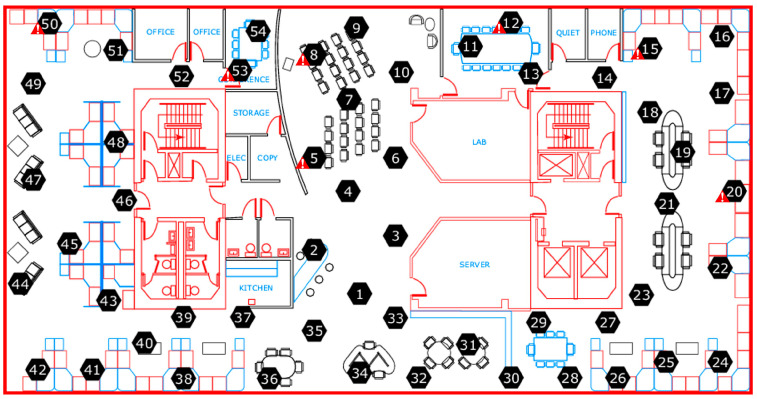
Distribution map of the sensors within the Intel lab (Note: The sensors with 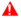 are abnormal sensors).

Through preliminary screening of the data, the abnormal sensors marked in Figure 2 were removed in the experiment. The temperature and humidity data collected by the remaining 47 sensors after one hour were selected for simulation, and it is considered that the entire wireless sensor network was in a stable state at this time. It is assumed that all nodes send data to a common CH located at the center of the laboratory, with coordinates (20.5, 15.5).

#### 3.2.1. Comparison Results of Compression Ratios at the Sensor Level

In this study, Algorithm 1 is deployed on each individual sensor device and subsequently juxtaposed with the pre-existing and relevant technique, namely, the IBE method. Due consideration is given to the inherent characteristics of the dataset. Consequently, the number of segments employed in the experimental setup is deliberately selected as a multiple of two. Specifically, when τ=32, v=2, and when τ=64, v=4. The requisite calculations are performed in accordance with Equation (13).

Recognizing that a solitary sample can be unduly influenced by the idiosyncrasies of the specific dataset, a comprehensive approach is adopted to assess the performance of Algorithm 1. To this end, the compression ratios are computed for a series of consecutive cycles during the experiment. Subsequently, the mean value of all these calculated compression ratios is determined.

**Figure 3:**
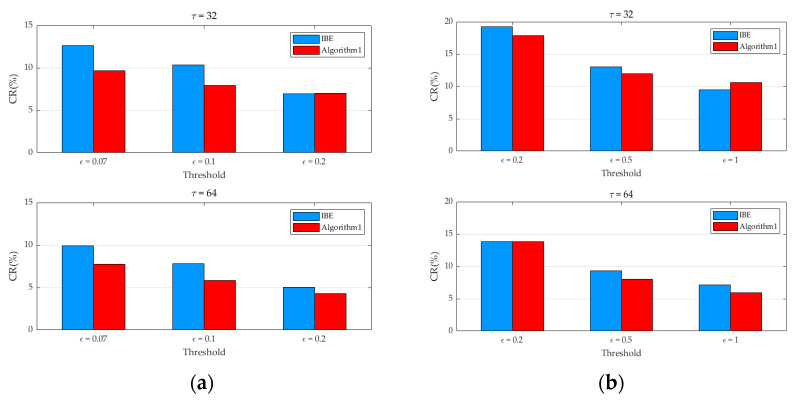
The relationship between the compression ratio of the algorithm and the threshold for different cycles. (**a**) Compression ratios of two algorithms for temperature data; (**b**) Compression ratios of two algorithms for humidity data.

It is important to note that the outcomes of the sparse vector compression algorithm predicated on segmented regions exhibit a high degree of correlation with both the cycle size and the similarity threshold. As depicted in Figure 3, the average compression ratios for 10 consecutive sets of temperature and humidity data are presented, considering various thresholds and cycle sizes.

The experimental findings reveal that, in nearly all instances, the compression ratio achieved by Algorithm 1 is lower than that of the IBE method. Even under the most unfavorable circumstances, the compression ratio of Algorithm 1 is only marginally 1.137% higher than that of the IBE method. Moreover, a distinct trend is observable: as the cycle size increases, the compression ratio of Algorithm 1 decreases. This phenomenon can be attributed to the fact that when the time interval is relatively short, the disparity between the data points and the key values of their respective segments remains relatively stable.

We calculated the average computation time for the data compression of the humidity data over ten cycles. The results show that the average computation time of the SV-ZIZO method is 0.0110 s, and that of the ZIZO method is 0.0107 s. There is little difference in the time cost between the two methods.

#### 3.2.2. Comparison Results of Reconstruction Accuracy at the CH Level

**Figure 4:**
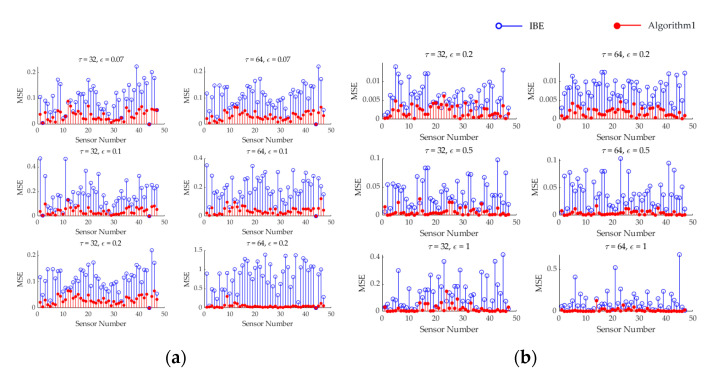
Comparison of the reconstruction accuracy of the algorithm under different cycles and different thresholds. (**a**) Mean squared errors of two algorithms for temperature data; (**b**) Mean squared errors of two algorithms for humidity data.

Taking the selected first cycle $S_{i}^{1}$ as an example, calculated according to Equation (14), it can be seen in Figure 4 that the reconstruction accuracy of the IBE algorithm is between (0, 1.4], while the reconstruction accuracy of Algorithm 1 is between (0, 0.25]. In almost all states under different thresholds and different cycles, the algorithm proposed in this paper exhibits higher reconstruction accuracy compared to the IBE method. And we found that as the threshold increases, the data recovery accuracy decreases, which is in line with the expected results.

#### 3.2.3. Adjustment Results of Dormant Sampling at the CH Level

At the CH, the spatial correlation degree of one cycle of data is calculated using the method in Section 2.2.3 to select some redundant nodes to enter the sleep state. The CH uniformly adjusts the sensing frequency of the enabled sensors in the cluster according to the temporal correlation degree of each cycle so as to try to minimize the collection of redundant data within the upper and lower bounds. The similarity level of one sensor’s readings can be offset by the difference level of another sensor’s readings in the cluster, resulting in the sampling rate being neither too high nor too low [18].

**Figure 5:**
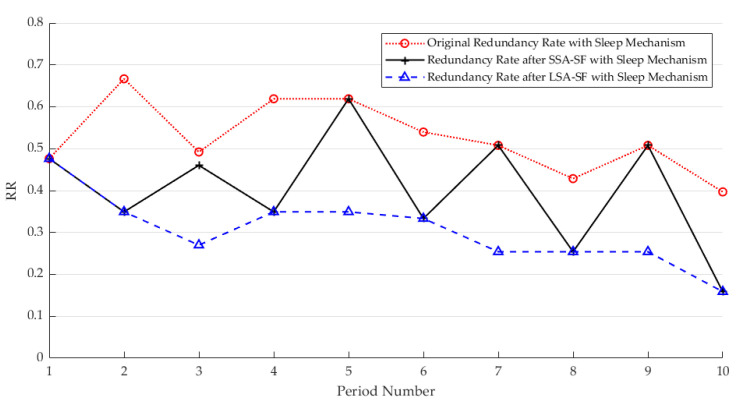
Effect of different sample rate adjustments on redundancy rates using a dormant mechanism.

The main purpose of adjusting the sampling frequency of sensors is to use correlation to discover redundancy in data and minimize the adjustment of the sampling frequency to further reduce redundant data. Taking temperature data as an example, here, λ=0.9995 is selected, the sensors are divided into 40 classes, and the combinations of sensors that enter the dormant state are 7, 21, 30, 36, 40, 42, 46, and 47. Figure 5 shows the change in the redundancy rate with the change in cycles after selecting the critical value to adjust the sampling rate under the sleep mechanism. It can be seen that the raw data collected continuously have a very high redundancy rate. Therefore, it is very important to select appropriate sampling rate adjustment parameters while ensuring data accuracy.

Here, we select the same data as in Section 3.2.1. It is recorded that the average running time of the SV-ZIZO method for data decompression and the CH-level processing proposed in this paper is 0.0558 s. The average running time of the ZIZO method for data decompression and CH-level processing is 0.0469 s. This is because the SV-ZIZO method consumes a certain amount of time in screening sensors using the fuzzy theory. However, overall, from the perspective of time cost, the gap is not significant.

#### 3.2.4. Comparison Results of Total Network Energy Consumption

One of the most important challenges faced by wireless sensor networks is reducing network energy consumption. This paper employs the Heinzelman model [22] to evaluate network energy consumption under different scenarios. In this model, energy consumption is mainly used for data transmission and reception in the network, and other factors (sensing, processing, etc.) are not considered for the time being. The data selection is the same as in Section 3.2.1. For each scenario, we calculated the ratio of the total energy consumption for transmitting data and the CH receiving data to the total energy consumption of transmitting and receiving data without using any method. Specifically, the scenarios included using Algorithm 1 alone, using Algorithm 1 with a large-scale adjustment of sampling frequency with a sleep mechanism (SV-ZIZO(LSA-SF)), using Algorithm 1 with a small-scale adjustment of sampling frequency with a sleep mechanism (SV-ZIZO(SSA-SF)), using IBE, using IBE with a large-scale adjustment of sampling frequency (ZIZO(LSA-SF)), and using IBE with a small-scale adjustment of sampling frequency (ZIZO(SSA-SF)), while changing the cycle size and the similarity threshold at the same time.

As can be seen in Table 3, under conditions of different cycles and thresholds, the energy consumption ratios of six algorithms for two data types are shown. It is evident that the SV-ZIZO method proposed in this paper can significantly reduce energy consumption in most cases, with the minimum energy consumption reaching 3.43%. Although the energy consumption of using Algorithm 1 alone is relatively high due to the improvement in data accuracy, combining it with the algorithm at the cluster-head (CH) level can effectively improve the energy consumption ratio.

### 3.3. LUCE Dataset and Result Analysis

The LUCE dataset is the result of a measurement experiment carried out by the Swiss Federal Institute of Technology in Lausanne to understand meteorological changes in urban environments. The experiment used a network of 92 wireless weather stations with the aim of obtaining comprehensive and detailed information on meteorological changes in urban environments, with a sampling interval of 30 s.

**Figure 6:**
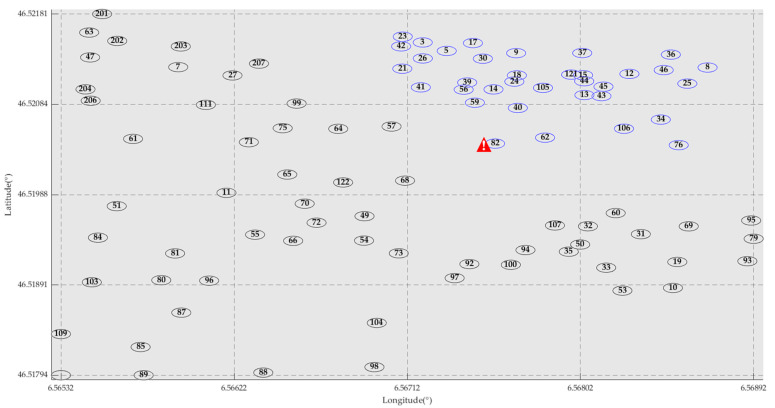
Latitude and Longitude Coordinate Diagram of LUCE Sensors (Note: The sensors with 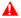 are abnormal sensors).

Considering the distance of outdoor sensors, this paper selects 35 sensors as indicated by the blue circles in Figure 6. Sensor No. 82 malfunctioned during the data collection process and was excluded from the experiment. Similarly, the temperature and humidity data collected by the remaining 34 sensors were simulated separately. Assume that all nodes send data to the central location of the selected area; Latitude: 46.5211°, Longitude: 6.5678°.

#### 3.3.1. Comparison Results of Compression Ratios at the Sensor Level

**Figure 7:**
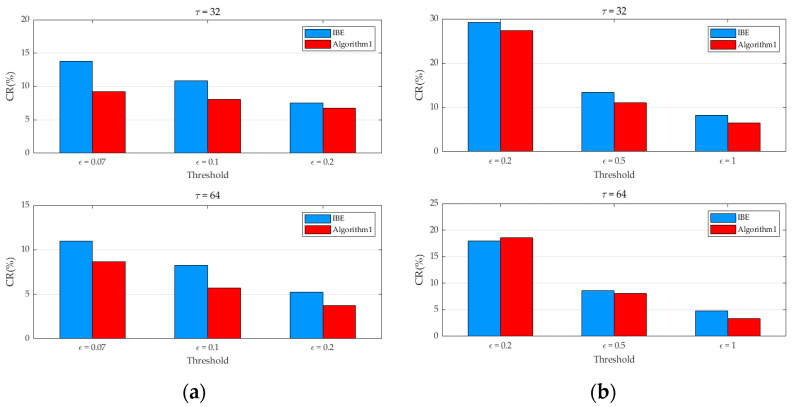
The relationship between the compression ratio of the algorithm and the threshold for different cycles. (**a**) Compression ratios of two algorithms for temperature data; (**b**) Compression ratios of two algorithms for humidity data.

In this section, an identical approach to that employed in Section 3.2.1 is utilized to compute the compression ratios of Algorithm 1 and the IBE algorithm for the genuine temperature and humidity data in the real-world environment. As depicted in Figure 7, the outcomes suggest that comparable conclusions to those presented in the previous figure can be derived. In nearly all instances, the compression ratio of Algorithm 1 is lower than that of the IBE method. Under the most unfavorable circumstances, the compression ratio of Algorithm 1 is merely 0.625% greater than that of the IBE method. Simultaneously, it can be noted that the compression ratio progressively diminishes as the threshold rises.

We also calculated the average computation time for the data compression of ten cycles in the real-world humidity data. The results show that the average computation time of the SV-ZIZO method is 0.0117 s, and that of the ZIZO method is 0.0092 s. The difference between the two is relatively small, which is in line with expectations.

#### 3.3.2. Comparison Results of Reconstruction Accuracy at the CH Level

**Figure 8:**
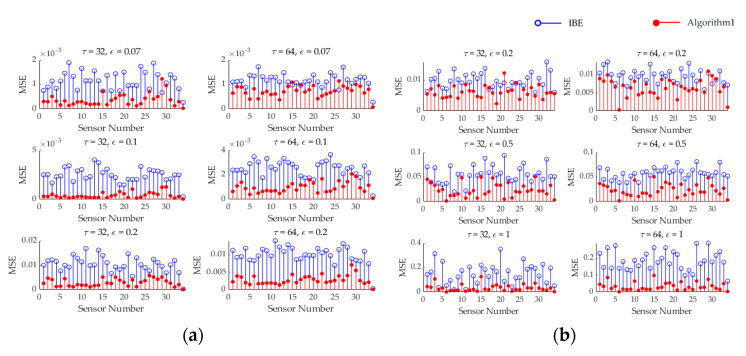
Comparison of the reconstruction accuracy of the algorithm under different cycles and different thresholds. (**a**) Mean squared errors of two algorithms for temperature data; (**b**) Mean squared errors of two algorithms for humidity data.

As can be seen in Figure 8, for this real-world dataset, the reconstruction accuracy of the IBE algorithm is in the range of (0, 0.35], while that of Algorithm 1 is in the range of (0, 0.13]. In almost all states, the algorithm proposed in this paper has better reconstruction accuracy than the IBE method. And we reach the same conclusion as that drawn from Figure 4.

#### 3.3.3. Adjustment Results of Dormant Sampling at the CH Level

This part applies to the temperature data in a real-world environment. At the cluster head (CH), the method described in Section 2.2.3 is used to calculate the spatial correlation of the data for one cycle so as to select some redundant nodes to enter the dormant state. Here, λ=0.995 is selected, the sensors are divided into 17 classes, and the combination of sensors entering the dormant state includes sensors numbered 9, 12, 16, 19, 26, 28, and 34.

**Figure 9:**
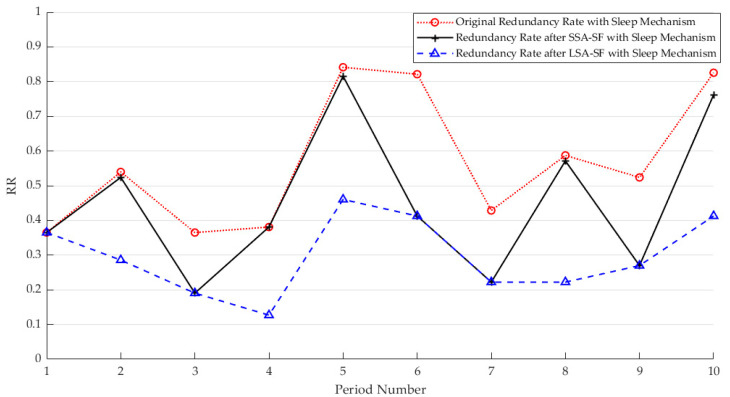
Effect of different sample rate adjustments on redundancy rates using a dormant mechanism.

The CH uniformly adjusts the sensing frequencies of the enabled sensors in the cluster according to the temporal correlation of each cycle, aiming to minimize the collection of redundant data within the upper and lower bounds. From the Figure 9, it can be observed that there is temporal redundancy in the sensor data. Therefore, it is necessary to adjust the sampling rates of the sensors. It is consistent with the results in Section 3.2.3.

The humidity data in the real environment show that the average running time for the data recovery of SV-ZIZO and the processing at the CH level is 0.0507 s, and the average running time for the data recovery of ZIZO and the processing at the CH level is 0.0459 s. The difference in results is acceptable.

#### 3.3.4. Comparison Results of Total Network Energy Consumption

In this section, we use the same calculation method as in Section 3.2.4. The energy consumption results of applying six algorithms to the temperature and humidity data in the real-world dataset are presented in Table 4. From this table, it can be seen that the energy consumption of the SV-ZIZO method proposed in this paper can reach a minimum of 2.35%. We also draw the same conclusion as in Table 3. That is, although the energy consumption of using Algorithm 1 alone is relatively high due to the improvement in data accuracy, combining it with the algorithm at the cluster-head (CH) level can effectively improve the energy consumption ratio.

## 4. Conclusions

To further improve the data compression ratio of existing methods while taking into account the problem of data reconstruction accuracy caused by data reduction, this paper proposes a data redundancy minimization algorithm based on sparse vectors. After the monitoring equipment stabilizes, a segmentation method is adopted at the sensor level, and the sparse vector compression method is used in combination with quartiles to compress the data of the first cycle of all sensors in the cluster. After uploading to the CH node, the spatial correlation of the reconstructed data matrix is considered, and sensors with high spatial redundancy are put into sleep mode using fuzzy theory knowledge. The redundancy rate of each cycle is calculated based on the temporal correlation of the data matrix of non-sleeping sensors, and the sampling rate of the sensors in the next cycle is adjusted in reverse. The experimental results show that the SV-ZIZO algorithm maximally reduces redundant data transmission while ensuring data accuracy and significantly improves energy consumption efficiency.

In future work, the method proposed in this paper can be improved in several directions. First, an adaptive threshold could be introduced to improve the compression mechanism. Because the actual application scenarios are complex and changeable, and the data characteristics also change dynamically, our goal is to solve problems such as the insufficient or excessive compression of some data that may be caused by fixed thresholds. Second, the dormancy strategy at the CH level still needs further exploration to maximize resource utilization and further extend the network lifespan. Finally, in view of the characteristics of different application scenarios in the actual Internet of Things, the feasibility of SV-ZIZO needs to be further explored.

## Figures and Tables

**Table 1:** Sampling rate adjustment table.

Redundancy Rate (RR)	Large-Scale Adjustment of Sampling Frequency (LSA-SF)	Small-Scale Adjustment of Sampling Frequency (SSA-SF)
0 ≤ RR ≤ 0.4	60%	100%
0.4 < RR ≤ 0.7	40%	60%
0.7 < RR ≤ 1	20%	40%

**Table 2:** Parameter setting.

Parameter	Values
*N*	IBRL: 47 LUCE: 34
Τ	32, 64
$E_{elec}$	50 nJ/bit
$\beta_{amp}$	100 pJ/bit/m^2^
*T*	0.2
Ε	Temperature: 0.07, 0.1, 0.2 Humidity: 0.2, 0.5, 1

**Table 3:** Comparison of energy consumption ratios of temperature and humidity data under six algorithms.

Data Type	Period	Threshold	Algorithm 1	SV-ZIZO(LSA-SF)	SV-ZIZO(SSA-SF)	IBE	ZIZO(LSA-SF)	ZIZO(SSA-SF)
Temperature	τ=32	ε=0.07	15.53%	10.11%	10.70%	12.95%	12.59%	12.65%
		ε=0.1	12.10%	7.37%	7.63%	10.41%	10.13%	10.24%
		ε=0.2	9.97%	6.28%	6.55%	6.90%	6.87%	6.88%
	τ=64	ε=0.07	12.29%	9.80%	10.99%	9.25%	9.01%	9.19%
		ε=0.1	9.09%	5.95%	6.56%	7.26%	7.09%	7.16%
		ε=0.2	6.33%	4.12%	4.36%	4.64%	4.59%	4.63%
Humidity	τ=32	ε=0.2	20.74%	5.56%	6.96%	19.23%	7.94%	8.65%
		ε=0.5	14.49%	4.06%	5.02%	12.18%	5.19%	5.52%
		ε=1	11.86%	3.43%	4.05%	9.74%	4.35%	4.54%
	τ=64	ε=0.2	17.62%	13.55%	13.66%	14.22%	11.29%	12.41%
		ε=0.5	11.36%	6.09%	7.37%	8.35%	6.77%	7.87%
		ε=1	8.72%	4.68%	5.62%	6.23%	5.20%	5.86%

**Table 4:** Comparison of energy consumption ratios of temperature and humidity data under six algorithms.

Data Type	Period	Threshold	Algorithm 1	SV-ZIZO (LSA-SF)	SV-ZIZO (SSA-SF)	IBE	ZIZO (LSA-SF)	ZIZO (SSA-SF)
Temperature	τ=32	ε=0.07	11.85%	3.48%	3.66%	17.16%	6.84%	6.86%
		ε=0.1	8.94%	3.03%	3.22%	13.35%	5.51%	5.62%
		ε=0.2	6.71%	2.62%	2.67%	8.78%	3.91%	3.94%
	τ=64	ε=0.07	8.72%	6.55%	7.07%	12.96%	11.95%	12.46%
		ε=0.1	5.81%	4.52%	4.72%	9.69%	9.19%	9.44%
		ε=0.2	3.56%	2.85%	2.93%	5.99%	5.71%	5.81%
Humidity	τ=32	ε=0.2	28.75%	13.24%	15.72%	29.66%	12.28%	13.43%
		ε=0.5	11.60%	4.29%	4.50%	14.62%	6.31%	6.61%
		ε=1	6.06%	2.78%	2.89%	8.65%	3.87%	4.02%
	τ=64	ε=0.2	25.62%	17.85%	18.85%	19.68%	17.95%	19.41%
		ε=0.5	8.47%	6.27%	6.58%	9.38%	8.86%	9.13%
		ε=1	3.48%	2.35%	2.85%	5.45%	5.22%	5.35%

## Data Availability

http://db.csail.mit.edu/labdata/labdata.html (accessed on 10 October 2023).
